# Supplementary material for: Presence of Multiple Genetic Mutations Related to Insecticide Resistance in Chinese Field Samples of Two Phthorimaea Pest Species
Source: Insects. 2024 Mar 14;15(3):194. doi: 10.3390/insects15030194 (PMC10971181; doi:10.3390/insects15030194)
Supplement: Supplementary file 1 [file insects-15-00194-s001.zip › TableS1.pdf]

Table S1.1 Primers for species identification

Gene	Primer	Sequences	Amplicon length
COI	LCO1490F	5’-GGTCAACAAATCATAAAGATATTGG-3’	~700bp
	HCO2198	5'-TAAACTTCAGGGTGACCAAAAAATCA-3'	

Table S1.2 Primers for sequence amplification

Gene	Primer	Sequence	Amplicon length
VGSC	*PoVGSC*-F	5'-TCATTGTGGCCTTATCACTGTTGGA-3'	~2500bp
	PoVGSC-R	5'-GTAACAGAGCTAAGAAGAGATTAAGTAC-3'	
	PaVGSC-F	5'-AATTGCAGCTTCGAGTATTCAAATTGG-3'	~1600bp
	PaVGSC-R	5'-GGGAGCGTGTATTGCTATCTGGT-3'	
Ace1	*PoAce1*-F	5'-ATGCGCGTAGTGTTGGCCGCCCT-3'	~3500bp
	PoAce1-R	5'-TCATATGATGTACTTAGACAGCATTGAAT-3'	
	PaAce1-F	5'-ATGCGCGTAGTGTTGGCCGCCC-3'	~3500bp
	PaAce1-R	5'-TCATATGATGTACTTAGACAGCATA-3'	
RDL	*PoRdl*-F	5'-GAAATTACTCAAGACTGGCTTGTGAG-3'	~650bp
	PoRdl-R	5'-CGAGTAGCTTACCGAAGGTCTACC-3'	

PaRdl-F	5’-GAAATTACTCAAGACTGGCTTGTGA-3’	~1000bp
PaRdl-R	5’-CGAAGGTCTACCGGGTGGA-3’	

Table S1.3 Primers for sequencing

Gene	Primer	Sequence
VGSC	*PoVGSC*-intron18F	5'-CACGTTATAAAGCTATGCCACGCAG-3'
	PoVGSC-exon21R	5'-GTAACAGAGCTAAGAAGAGATTAAGTAC-3'
	PaVGSC-exon17F	5'-AATTGCAGCTTCGAGTATTCAAATTGG-3'
	PaVGSC-exon18F	5'-CGTTGATCGTTTTCCCGATGGAG-3'
Ace1	*PoAce1*-exon1F	5'-CCGAACACAGATATGCAGGAAGAC-3'
	PaAce1-exon1F	5'-TTCCCGGCGCGATGATGTGGAATC-3'
RDL	*PoRdl*-F	5'-GAAATTACTCAAGACTGGCTTGTGAG-3'
	PaRdl-F	5'-GAAATTACTCAAGACTGGCTTGTGA-3'
