# Supplementary material for: Presence of Multiple Genetic Mutations Related to Insecticide Resistance in Chinese Field Samples of Two Phthorimaea Pest Species
Source: Insects. 2024 Mar 14;15(3):194. doi: 10.3390/insects15030194 (PMC10971181; doi:10.3390/insects15030194)
Supplement: Supplementary file 1 [file insects-15-00194-s001.zip › TableS2.pdf]

TableS2.1 Sequence information for the divergence time analysis

Types	Species	GenBank Accession Numbers
the COI gene of *Gelechiidae*	*Scrobipalpa costella*	OX463325.1
	Teleiodes luculella	OX419612.1
	Keiferia lycopersicella	JN305836.1
	Phthorimaea operculella	NC_057501.1
	Scrobipalpa atriplicella	JARACY010001406.1
	Tuta absoluta	NC_050874.1
	Pectinophora gossypiella	KM225795.1
	Carpatolechia fugitivella	OX579675.1
	Athrips mouffetella	OX383987.1

	Aproaerema taeniolella	OX465273.1
	Anarsia innoxiella	OX387421.1
	Plutella xylostella	NC_025322.1
	Spodoptera frugiperda	NC_027836.1
	Helicoverpa armigera	NC_014668.1
	Drosophila melanogaster	AF200828.1

TableS2.2 Sequence information for haplotypes

Types	Species	GenBank Accession Numbers
the haplotype of *VGSC*	*PoVGSC*exon19-H1	OR963514
	*PoVGSC*exon20-H1	OR963515
	*PoVGSC*exon20-H2	OR963516
	*PoVGSC*exon20-H3	OR963517
	*PaVGSC*exon17-H1	OR963518
	*PaVGSC*exon17-H2	OR963519
	*PaVGSC*exon17-H3	OR963520
	*PaVGSC*exon17-H4	OR963521
	*PaVGSC*exon17-H5	OR963522
	*PaVGSC*exon17-H6	OR963523

	*PaVGSC*exon17-H7	OR963524
	*PaVGSC*exon17-H8	OR963525
	*PaVGSC*exon17-H9	OR963526
	*PaVGSC*exon18-H1	OR963527
the haplotype of *Ace1*	*PoAce1*-H1	OR963498
	PoAce1-H2	OR963499
	PoAce1-H3	OR963500
	PoAce1-H4	OR963501
	PoAce1-H5	OR963502
	PoAce1-H6	OR963503
	PaAce1-H1	OR963504

	PaAce1-H2	OR963505
	PaAce1-H3	OR963506
	PaAce1-H4	OR963507
the haplotype of *RDL*	*PoRDL*-H1	OR963508
	PaRDL-H1	OR963509
	PaRDL-H2	OR963510
	PaRDL-H3	OR963511
	PaRDL-H4	OR963512
	PaRDL-H5	OR963513
