# Supplementary material for: Presence of Multiple Genetic Mutations Related to Insecticide Resistance in Chinese Field Samples of Two Phthorimaea Pest Species
Source: Insects. 2024 Mar 14;15(3):194. doi: 10.3390/insects15030194 (PMC10971181; doi:10.3390/insects15030194)
Supplement: Supplementary file 1 [file insects-15-00194-s001.zip › TableS3.pdf]

TableS3 Informations on field strain of *P. operculella* and *P. absoluta* used in the present study. All strains, except XS strain from tomato plants, were collected from potato plants.

Species	Strain	Collection location	Coordinate	Date	Stage
Phthorimaea operculella	ML	Malong District, Qujing City	103º34'50"；25º16'24"	April, 2023	Larva
	LL	Luliang County, Qujing City	103º40'48"；25º9'51"	April, 2023	Larva
	XW	Xuanwei City, Qujing City	104º04'28″；26º05'52″	August, 2023	Larva
Phthorimaea absoluta	PL	Panlong District, Kunming City	102º44'56″；25º7'42″	April, 2023	Larva
	GD	Guandu District, Kunming City	102º56'51"；25º10'40″	April, 2023	Larva
	XS	Xinshi District, Urumqi City	87º29'47"；43º57'6″	August, 2023	Larva
